# Methodology for extracting activity from functional calcium imaging data

**DOI:** 10.1186/1471-2202-13-S1-P173

**Published:** 2012-07-16

**Authors:** Allison Del Giorno, Natalia Toporikova, Nick Mellen, Robert J Butera

**Affiliations:** 1 Electrical Engineering, Georgia Institute of Technology, Atlanta, Georgia 30332, USA; 2 NeuroLab, Georgia Institute of Technology, Atlanta, Georgia, 30332, USA; 3 Kosair Children's Hospital Research Institute, University of Louisville, Louisville, Kentucky, 40202, USA

## 

**Figure 1:**
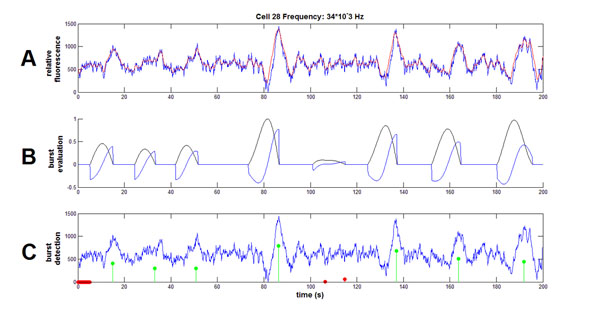
Steps in extraction of activity episodes. Calculated frequency (34 kHz) is displayed in the figure heading. **A.** Sample trace (blue) and smoothed result of first Butterworth filter (red). **B.** Modified first derivative (black) and subsequent negated second derivative (blue). **C.** Identification of candidates that are local minima of second derivative (red, green) and final selected locations of activity episodes (green).

Functional calcium imaging is a widespread technique used to measure the activity of multiple cells at a time. Because the activity traces from this data collection technique are noisy, we have developed a new method for extraction of activity episodes from the fluorescence traces. While Gaussian filters are commonly used for smoothing data, this technique alters the frequency of the activity episodes, so instead Butterworth filters are employed. We plotted the spectrograms of individual traces and visually determined the cutoff frequency that would remove the high-frequency noise. After smoothing (Figure 1A), we identify local minima by taking a modified first derivative and then calculating the second derivative from this trace (Figure 1B). Local minima are then selected as activity episodes based on their corresponding first derivative values (Figure 1C). These activity locations are then used in the calculations for frequency of activity episodes. This method has several advantages for use with calcium data: (1) it is independent of the amplitude of the bursts; (2) the function is robust to drift in the trace; (3) the parameters are easily changed to adapt to ground truth data under differing experimental conditions.

